# Graphene Oxide Dysregulates Neuroligin/NLG-1-Mediated Molecular Signaling in Interneurons in *Caenorhabditis elegans*

**DOI:** 10.1038/srep41655

**Published:** 2017-01-27

**Authors:** He Chen, Huirong Li, Dayong Wang

**Affiliations:** 1 Key Laboratory of Environmental Medicine Engineering in Ministry of Education, Medical School, Southeast University, Nanjing 210009, China

## Abstract

Graphene oxide (GO) can be potentially used in many medical and industrial fields. Using assay system of *Caenorhabditis elegans*, we identified the NLG-1/Neuroligin-mediated neuronal signaling dysregulated by GO exposure. In nematodes, GO exposure significantly decreased the expression of NLG-1, a postsynaptic cell adhesion protein. Loss-of-function mutation of *nlg-1* gene resulted in a susceptible property of nematodes to GO toxicity. Rescue experiments suggested that NLG-1 could act in AIY interneurons to regulate the response to GO exposure. In the AIY interneurons, PKC-1, a serine/threonine protein kinase C (PKC) protein, was identified as the downstream target for NLG-1 in the regulation of response to GO exposure. LIN-45, a Raf protein in ERK signaling pathway, was further identified as the downstream target for PKC-1 in the regulation of response to GO exposure. Therefore, GO may dysregulate NLG-1-mediated molecular signaling in the interneurons, and a neuronal signaling cascade of NLG-1-PKC-1-LIN-45 was raised to be required for the control of response to GO exposure. More importantly, intestinal RNAi knockdown of *daf-16* gene encoding a FOXO transcriptional factor in insulin signaling pathway suppressed the resistant property of nematodes overexpressing NLG-1 to GO toxicity, suggesting the possible link between neuronal NLG-1 signaling and intestinal insulin signaling in the regulation of response to GO exposure.

Graphene family, 2D carbon engineered nanomaterials (ENMs), have attracted massive attention due to its unique mechanical, electronic, and thermal properties^1^. Graphene family, including graphene oxide (GO), can be potentially used in many fields, especially in the catalysis, the biosensor, and the biomedicine^2^. Meanwhile, so far, the evidence from both *in vitro* and *in vivo* studies has demonstrated the possible toxicity of some members in the graphene family, such as GO, on organisms^3^,^4^,^5^,^6^,^7^. Moreover, the mechanisms of both genetic and epigenetic control of response to GO exposure have been examined in human cell lines, such as HepG2 cell and GLC-82 cell, and animals, such as mice^4^,^8^,^9^.

*Caenorhabditis elegans*, a classic model animal, has been widely used as an *in vivo* assay system for toxicological study of environmental toxicants^10^,^11^,^12^. *C. elegans* has the typical properties of model animals, and the properties at least include the short life-cycle, short lifespan, transparent body, self-fertilization, and ease of culture^13^. Using a series of sublethal endpoints, *C. elegans* has been successfully used in the toxicity assessment of many environmental toxicants including the ENMs^14^,^15^. Exposure to GO could potentially cause toxic effects on the functions of both primary targeted organs such as intestinal cells and secondary targeted organs such as neurons and reproductive organs in nematodes^16^,^17^,^18^,^19^,^20^,^21^. Some important signaling pathways, such as c-Jun N-terminal kinase (JNK), insulin, p38 mitogen-activated protein kinase (MAPK), Wnt, oxidative stress associated, apoptosis, and DNA damage signaling pathways, have been further identified to be involved in the regulation of GO toxicity in nematodes^22^,^23^,^24^,^25^,^26^,^27^,^28^,^29^,^30^. Besides these signaling pathways, some microRNAs (miRNAs), such as *mir-231* and *mir-360*, were also shown to participate in the control of GO toxicity in nematodes^23^,^26^,^31^.

In animals, besides the behaviors, the nervous system also regulates some other biological processes, such as longevity, fat storage, and stress response^32^,^33^. However, it is still largely unclear for the role of neuronal signals in the regulation of response to ENMs in animals. In *C. elegans, nlg-1* gene encodes a Neuroligin, a postsynaptic cell adhesion protein. Neuroligins can bind to presynaptic proteins like neurexins and act as central organizing molecules that connect pre- and post-synaptic neurons^34^. NLG-1 has been shown to be required for the control of synaptic function and oxidative stress in nematodes^35^,^36^,^37^. In the present study, using the *in vivo* assay system of *C. elegans*, we investigated the effect of GO exposure on NLG-1-mediated signaling in neurons in animals. Moreover, we examined the possible underlying molecular mechanism for the function of neuronal NLG-1 in the regulation of response to GO exposure. Our results provide the important molecular basis for NLG-1-mediated neuronal signaling in the regulation of response to GO exposure in organisms.

## Results

### Effects of GO exposure on expression of *nlg-1* gene

**Figure 1:**
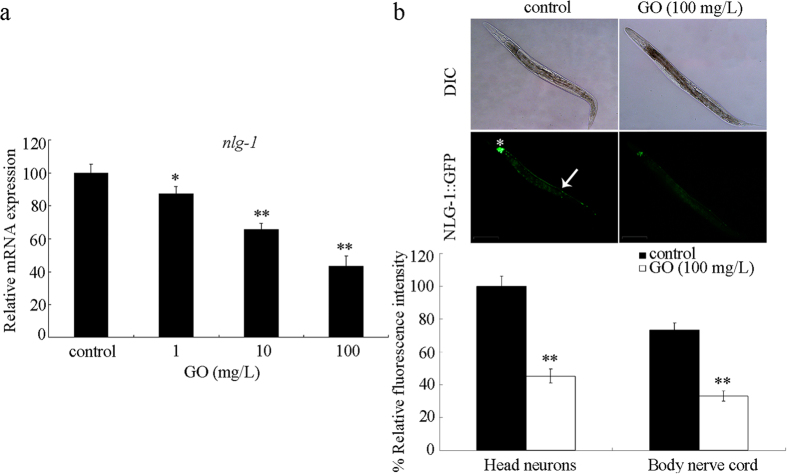
Effects of GO exposure on expression of *nlg-1* gene in wild-type nematodes. (**a**) Effects of GO exposure at different concentrations on transcriptional expression of *nlg-1* gene. (**b**) Effects of GO exposure on expression of NLG-1::GFP in neurons. Asterisk indicates the head neurons, and arrowhead indicates the body nerve cord. GO exposure concentration was 100 mg/L. Prolonged exposure was performed from L1-larvae to young adults. Bars represent means ± SD. **P < 0.01 *vs* control.

It was reported that GO at the concentration of 1 mg/L could cause the decrease in locomotion behavior, and the significant induction of ROS production in nematodes^17^. We selected the 1, 10, and 100 mg/L as the working concentrations for GO. After prolonged exposure from L1-larvae to young adults, GO at the concentrations of 10 and 100 mg/L significantly decreased the transcriptional expression of *nlg-1* gene in wild-type N2 nematodes (Fig. 1a). GO at the concentration of 1 mg/L moderately but significantly decreased the transcriptional expression of *nlg-1* gene in wild-type N2 nematodes (Fig. 1a). Moreover, GO at the concentration of 100 mg/L significantly reduced the expression of NLG-1::GFP in both the head neurons and the body nerve cord in wild-type N2 nematodes (Fig. 1b). Therefore, GO exposure may potentially decrease the NLG-1 expression in wild-type N2 nematodes.

### Mutation of *nlg-1* gene induced a susceptible property of nematodes to GO toxicity

**Figure 2:**
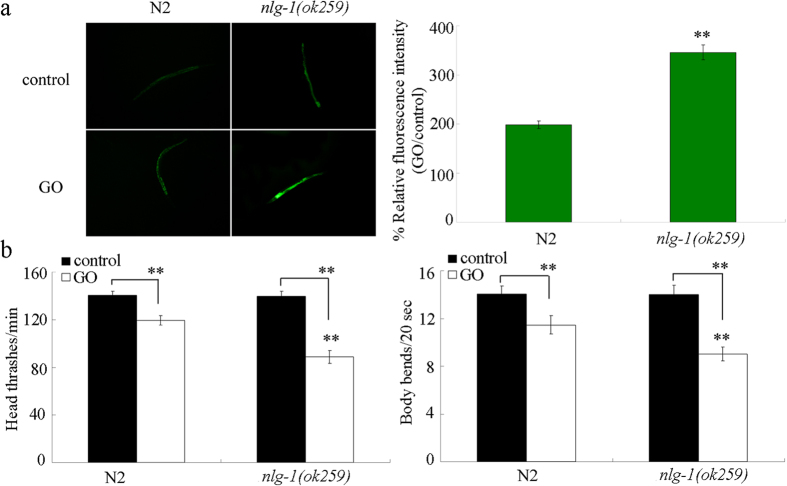
Mutation of *nlg-1* gene induced a susceptible property to GO toxicity. (**a**) Mutation of *nlg-1* gene induced a susceptible property to GO toxicity in inducing intestinal ROS production. (**b**) Mutation of *nlg-1* gene induced a susceptible property to GO toxicity in decreasing locomotion behavior. Prolonged exposure was performed from L1-larvae to young adults. GO exposure concentration was 1 mg/L. Bars represent means ± SD. **P < 0.01 *vs* N2 (if not specially indicated).

We next used the loss-of-function mutant of *nlg-1(ok259*) to investigate the effects of *nlg-1* mutation on the formation of GO toxicity. Under the normal condition, we detected an obvious induction of intestinal reactive oxygen species (ROS) production in *nlg-1(ok259*) mutant (Fig. 2a). After GO (1 mg/L) exposure, a more significant induction of intestinal ROS production was observed in *nlg-1(ok259*) mutant compared with that in wild-type N2 nematodes (Fig. 2a). Under the normal condition, we observed the normal locomotion behaviors of head thrash and body bend in *nlg-1(ok259*) mutant (Fig. 2b). After GO (1 mg/L) exposure, a significantly decreased head thrash or body bend was found in *nlg-1(ok259*) mutant compared with that in wild-type nematodes (Fig. 2b). Therefore, a susceptible property to GO toxicity may be formed in loss-of-function *nlg-1* mutant nematodes.

### Neuron-specific activities of NLG-1 in the regulation of response to GO exposure

**Figure 3:**
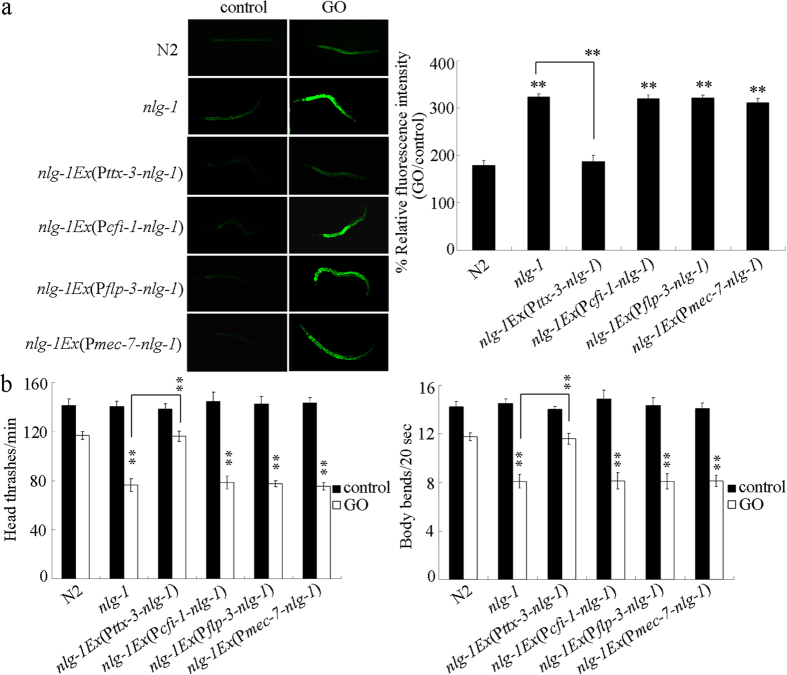
Neuron-specific activities of NLG-1 in regulating response to GO exposure. (**a**) Neuron-specific activities of NLG-1 in regulating GO toxicity in inducing intestinal ROS production. (**b**) Neuron-specific activities of NLG-1 in regulating GO toxicity in decreasing locomotion behavior. Prolonged exposure was performed from L1-larvae to young adults. GO exposure concentration was 1 mg/L. Bars represent means ± SD. **P < 0.01 *vs* N2 (if not specially indicated).

In *C. elegans*, NLG-1 is observed to be expressed in neurons, including PVD sensory neurons, URB and AIY interneurons, and URA motor neurons^28^. Using the corresponding promoter for AIY, URA, URB, or PVD neurons, we investigated the neuron-specific activities of NLG-1 in the regulation of response to GO exposure. After GO (1 mg/L) exposure, we found that expression of the NLG-1 in the AIY interneurons could suppress the susceptible property to GO toxicity in inducing intestinal ROS production and in decreasing locomotion behavior in *nlg-1(ok259*) mutant nematodes (Fig. 3). In contrast, after GO (1 mg/L) exposure, exposure of the NLG-1 in the PVD sensory neurons, the URB interneurons, or the URA motor neurons could not obviously affect the susceptible property to GO toxicity in inducing intestinal ROS production and in decreasing locomotion behavior in *nlg-1(ok259*) mutant nematodes (Fig. 3). These results suggest that NLG-1 can act in the AIY interneurons to regulate the response to GO exposure.

### Mutation of *nlg-1* gene dysregulated the expression of some genes expressed in the AIY interneurons in GO exposed nematodes

**Figure 4:**
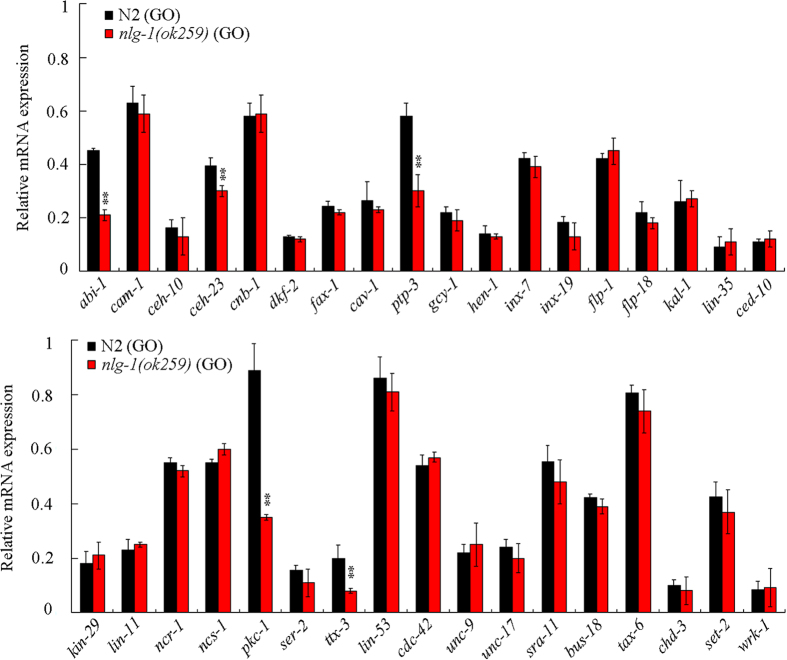
Effects of *nlg-1* mutation on expression patterns of genes expressed in AIY interneurons in GO exposed nematodes. Prolonged exposure was performed from L1-larvae to young adults. GO exposure concentration was 1 mg/L. Bars represent means ± SD. **P < 0.01 *vs* N2 (GO).

To determine the molecular targets for NLG-1 in AIY interneurons in the regulation of response to GO exposure, we investigated the expression patterns of genes expressed in the AIY interneurons (http://legacy.wormbase.org/db/searches/expr_search#results) in GO exposed wild-type and GO exposed *nlg-1(ok259*) mutant nematodes. After GO (1 mg/L) exposure, among the examined 35 genes expressed in the AIY interneurons, we found that mutation of *nlg-1* gene significantly decreased the expression levels of *abi-1, ceh-23, ptp-3, pkc-1*, and *ttx-3* (Fig. 4). In *C. elegans, abi-1* encodes an Abl interactor, *ceh-23* encodes a homeodomain protein, *ptp-3* encodes a receptor-like tyrosine phosphatase, *pkc-1* encodes a serine/threonine protein kinase C (PKC) protein, and *ttx-3* encodes a LIM homeodomain protein.

### PKC-1 was involved in the control of response to GO exposure

**Figure 5:**
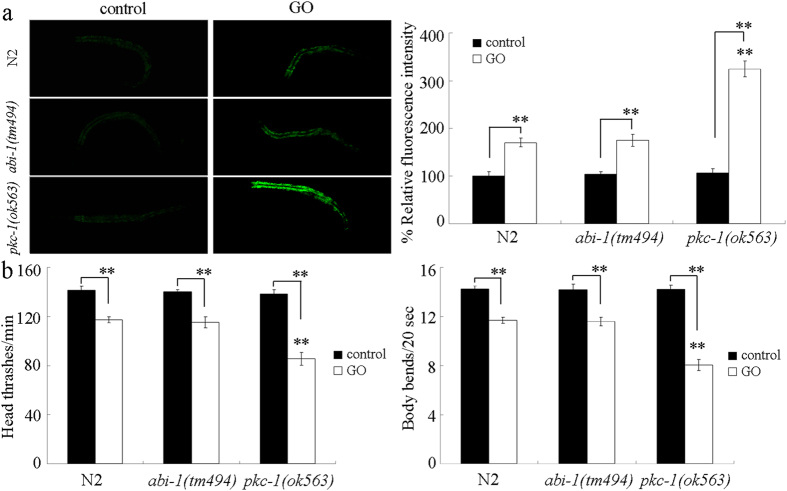
Effects of *abi-1* or *pkc-1* mutation on GO toxicity. (**a**) Effects of *abi-1* or *pkc-1* mutation on GO toxicity in inducing intestinal ROS production. (**b**) Effects of *abi-1* or *pkc-1* mutation on GO toxicity in decreasing locomotion behavior. Prolonged exposure was performed from L1-larvae to young adults. GO exposure concentration was 1 mg/L. Bars represent means ± SD. **P < 0.01 *vs* N2 (if not specially indicated).

Considering the fact that *ttx-3, ceh-23*, and *ptp-3* genes are required for the cell fate specification of AIY interneurons or the presynaptic differentiation in *C. elegans*^38^,^39^, we next examined whether ABI-1 and PKC-1 are involved in the control of response to GO exposure. PKC family plays critical roles in cell signaling in organisms^40^. In *C. elegans*, PKC-1 plays crucial roles in regulating the cell signaling and function of nervous system^41^,^42^. *abi-1* gene is required for the proper cell migration and apoptotic cell engulfment^43^,^44^. After GO (1 mg/L) exposure, we found that mutation of *pkc-1* gene induced a susceptible property to GO toxicity in inducing intestinal ROS production and in decreasing locomotion behavior (Fig. 5). In contrast, after GO (1 mg/L) exposure, mutation of *abi-1* gene did not obviously affect the GO toxicity in inducing intestinal ROS production and in decreasing locomotion behavior (Fig. 5). Therefore, our results suggest that PKC-1 may act a downstream target for NLG-1 in AIY interneurons to regulate the response to GO exposure.

### PKC-1 acted downstream of NLG-1 in the AIY interneurons to regulate the response to GO exposure

**Figure 6:**
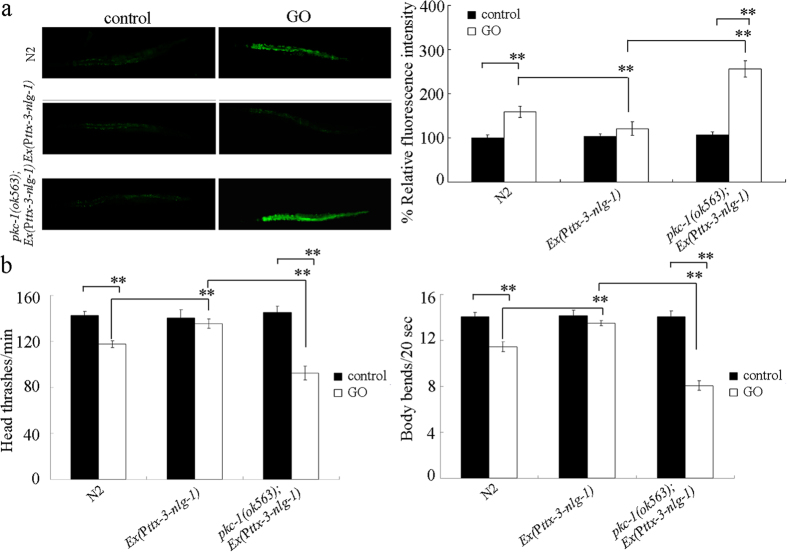
Effects of *pkc-1* mutation on the induction of intestinal ROS production (**a**) and the locomotion behavior (**b**) in GO exposed nematodes overexpressing *nlg-1* gene in AIY interneurons. Prolonged exposure was performed from L1-larvae to young adults. GO exposure concentration was 1 mg/L. Bars represent means ± SD. **P < 0.01.

To confirm whether PKC-1 could act downstream of NLG-1 in the AIY interneurons to regulate the response to GO exposure, *nlg-1* gene was overexpressed in the AIY interneurons of *pkc-1(ok563*) mutant nematodes. Using intestinal ROS production and locomotion behavior as the endpoints, we observed that overexpression of *nlg-1* gene in the AIY interneurons resulted in a resistant property to GO (1 mg/L) toxicity in inducing intestinal ROS production and in decreasing locomotion behavior (Fig. 6). However, after GO (1 mg/L) exposure, mutation of *pkc-1* gene obviously suppressed the resistant property of transgenic nematodes overexpressing NLG-1 in the AIY interneurons to GO toxicity in inducing intestinal ROS production and in decreasing locomotion behavior (Fig. 6). These results suggest that, in the AIY interneurons, PKC-1 can act downstream of NLG-1 to regulate the response to GO exposure.

### Identification of PKC-1-mediated signaling in the regulation of response to GO exposure

**Figure 7:**
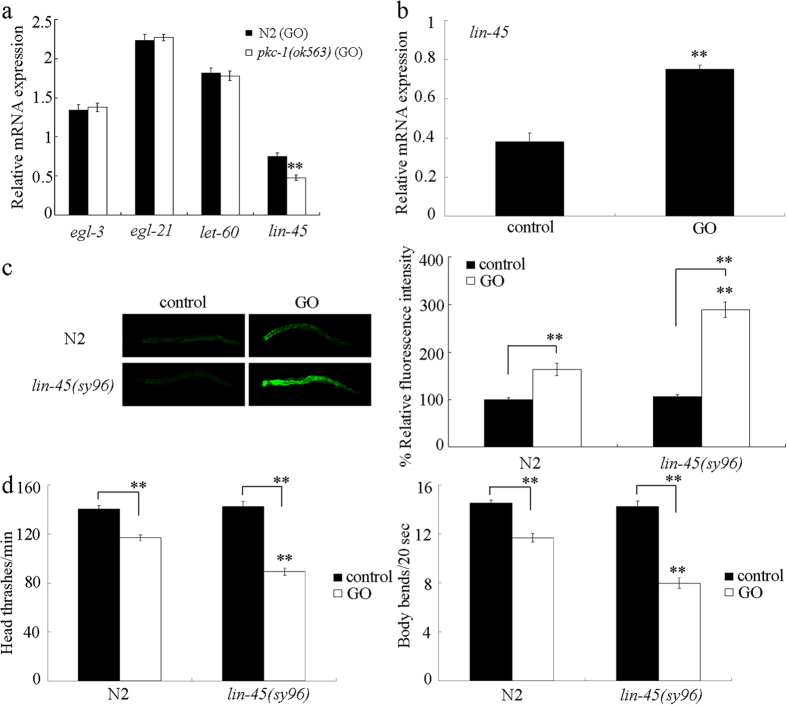
Identification of candidate targets for PKC-1 in regulating response to GO exposure. (**a**) Effects of *pkc-1* mutation on expression of *egl-3, egl-21, let-60*, and *lin-45* in GO exposed nematodes. Bars represent means ± SD. **P < 0.01 *vs* N2 (GO). (**b**) Effect of GO exposure on *lin-45* expression in wild-type nematodes. Bars represent means ± SD. **P < 0.01 *vs* control. (**c**) Effect of *lin-45* mutation on GO toxicity in inducing intestinal ROS production. Bars represent means ± SD. **P < 0.01 *vs* N2 (if not specially indicated). (**d**) Effect of *lin-45* mutation on GO toxicity in decreasing locomotion behavior. Bars represent means ± SD. **P < 0.01 *vs* N2 (if not specially indicated). Prolonged exposure was performed from L1-larvae to young adults. GO exposure concentration was 1 mg/L.

To further identify the functional targets for PKC-1 in the regulation of response to GO exposure, a subset of PKC-1 targets identified in *C. elegans* were examined^42^. Among the examined four candidate targeted genes, *egl-3* and *egl-21* genes encode two proneuropeptide processing enzymes, *let-60* gene encodes a Ras protein ERK signaling pathway, and *lin-45* gene encodes a Raf protein in ERK signaling pathway. After GO (1 mg/L) exposure, we found that *pkc-1* mutation significantly decreased the expression level of *lin-45* gene (Fig. 7a). In contrast, after GO (1 mg/L) exposure, we found that *pkc-1* mutation did not significantly affect the expression level of *egl-3, egl-21*, or *let-60* gene (Fig. 7a). Meanwhile, in wild-type N2 nematodes, GO (1 mg/L) exposure significantly increased the expression of *lin-45* gene (Fig. 7b). These results imply that *lin-45* may act as a functional targeted gene for *pkc-1* in the regulation of response to GO exposure.

### Mutation of *lin-45* induced a susceptible property to GO toxicity

Using intestinal ROS production and locomotion behavior as the endpoints, after GO (1 mg/L) exposure, we found that mutation of *lin-45* gene caused the formation of a susceptible property to GO toxicity in inducing intestinal ROS production and in decreasing locomotion behavior (Fig. 7c,d). Therefore, mutation of *lin-45* may induce a susceptible property to GO toxicity.

### LIN-45 acted downstream of PKC-1 in the AIY interneurons to regulate the response to GO exposure

**Figure 8:**
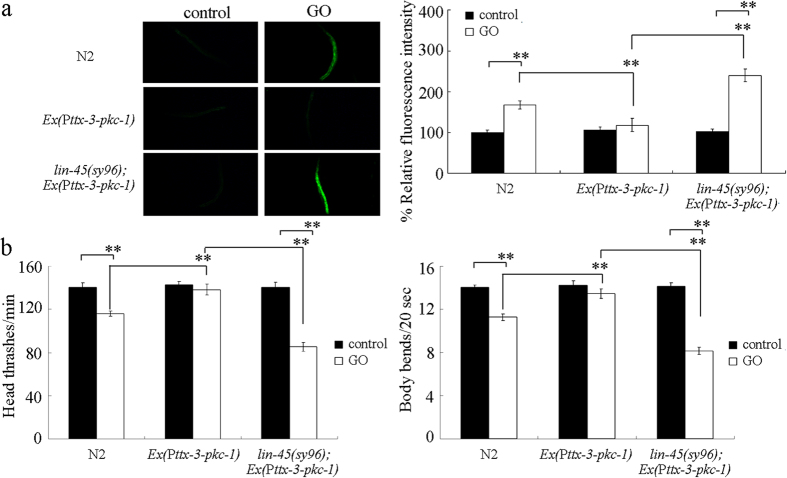
Effects of *lin-45* mutation on the induction of intestinal ROS production (**a**) and the locomotion behavior (**b**) in GO exposed nematodes overexpressing *pkc-1* gene in the AIY interneurons. Prolonged exposure was performed from L1-larvae to young adults. GO exposure concentration was 1 mg/L. Bars represent means ± SD. **P < 0.01.

To confirm whether LIN-45 could act downstream of PKC-1 in the AIY interneurons to regulate the response to GO exposure, *pkc-1* gene was overexpressed in the AIY interneurons in *lin-45(sy96*) mutant nematodes. Using intestinal ROS production and locomotion behavior as the endpoints, we observed that overexpression of *pkc-1* gene in the AIY interneurons induced in a resistant property to GO (1 mg/L) toxicity in inducing intestinal ROS production and in decreasing locomotion behavior (Fig. 8). However, after GO (1 mg/L) exposure, mutation of *lin-45* gene noticeably inhibited the resistant property of transgenic nematodes overexpressing PKC-1 in the AIY interneurons to GO toxicity in inducing intestinal ROS production and in decreasing locomotion behavior (Fig. 8). Therefore, LIN-45 may act downstream of PKC-1 in the AIY interneurons to regulate the response to GO exposure.

### PKC-1 and LIN-45 could act in the AIY interneurons to regulate response to GO exposure

**Figure 9:**
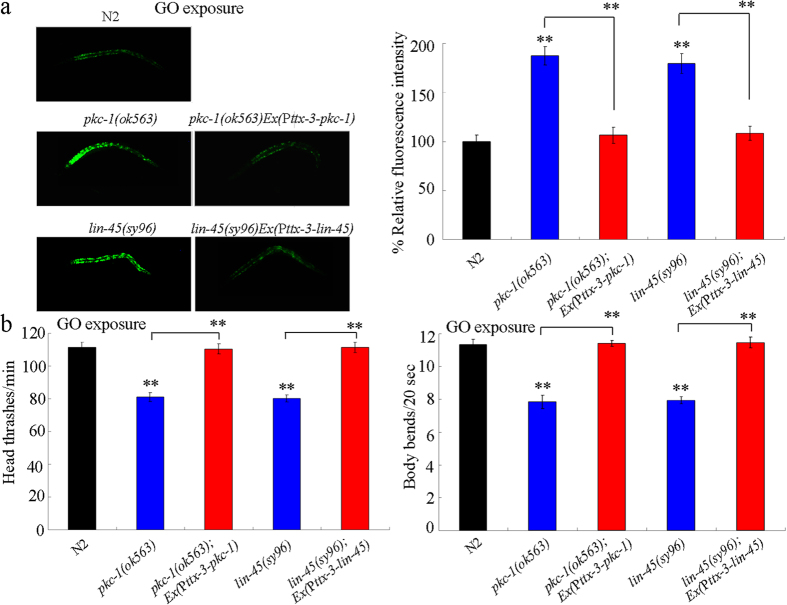
Rescue assay for the function of PKC-1 or LIN-45 expressed in the AIY interneurons in the regulation of response to GO exposure. (**a**) Rescue assay for the function of PKC-1 or LIN-45 expressed in the AIY interneurons in regulating GO toxicity in inducing intestinal ROS production. (**b**) Rescue assay for the function of PKC-1 or LIN-45 expressed in the AIY interneurons in regulating GO toxicity in decreasing locomotion behavior. Prolonged exposure was performed from L1-larvae to young adults. GO exposure concentration was 1 mg/L. Bars represent means ± SD. **P < 0.01 *vs* N2 (if not specially indicated).

In *C. elegans*, both *pkc-1* and *lin-45* are expressed in the neurons. After GO (1 mg/L) exposure, we found that expression of *pkc-1* gene in the AIY interneurons could effectively suppress the susceptible property of *pkc-1(ok563*) mutant to GO toxicity in inducing intestinal ROS production and in decreasing locomotion behavior (Fig. 9). Similarly, after GO (1 mg/L) exposure, expression of *lin-45* gene in the AIY interneurons could also significantly inhibit the susceptible property of *lin-45(sy96*) mutant to GO toxicity in inducing intestinal ROS production and in decreasing locomotion behavior (Fig. 9). These results imply that PKC-1 and LIN-45 can function in the AIY interneurons to regulate response to GO exposure.

### Distribution and translocation of GO in the body of *nlg-1, pkc-1*, and *lin-45* mutant nematodes

**Figure 10:**
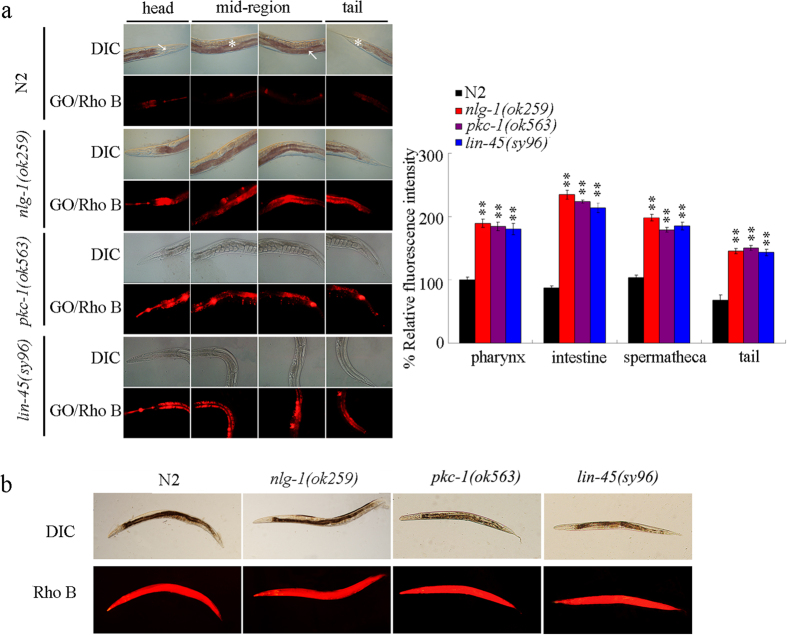
Distribution and translocation of GO in wild-type, *nlg-1(ok259*), *pkc-1(ok563*), and *lin-45(sy96*) mutant nematodes. (**a**) Distribution of GO/Rho B in wild-type, *nlg-1(ok259*), *pkc-1(ok563*), and *lin-45(sy96*) mutant nematodes. The arrowheads indicate the pharynx and intestine, respectively, in the head region or mid-region in nematodes. The spermatheca (*) in the mid-region and tail (**) are also indicated. Prolonged exposure was performed from L1-larvae to young adults. GO exposure concentration was 1 mg/L. Bars represent means ± SD. **P < 0.01 *vs* N2. (**b**) Distribution of Rho B in wild-type, *nlg-1(ok259*), *pkc-1(ok563*), and *lin-45(sy96*) mutant nematodes.

Distribution and translocation are crucial for the toxicity formation of certain ENMs including the GO in nematodes^17^,^45^. In this study, GO was labeled with molecular probe Rhodamine B (Rho B). After GO/Rho B (1 mg/L) exposure, we observed that mutation of *nlg-1, pkc-1*, or *lin-45* gene significantly increased the distribution of GO/Rho B in the pharynx, the intestine, the spermatheca, and the tail of nematodes compared with wild-type N2 nematodes (Fig. 10a). Compared with distribution of GO/Rho B, exposure to Rho B resulted in a relatively equable distribution of Rho B fluorescence in tissues of wild-type, *nlg-1(ok259*), *pkc-1(ok5630*), or *lin-45(sy96*) mutant nematodes (Fig. 10b). These data suggest that mutation of genes encoding the NLG-1-PKC-1-LIN-45 signaling pathway may affect both the toxicity and the translocation of GO.

### Genetic interaction between NLG-1 and DAF-16 in the regulation of response to GO exposure

**Figure 11:**
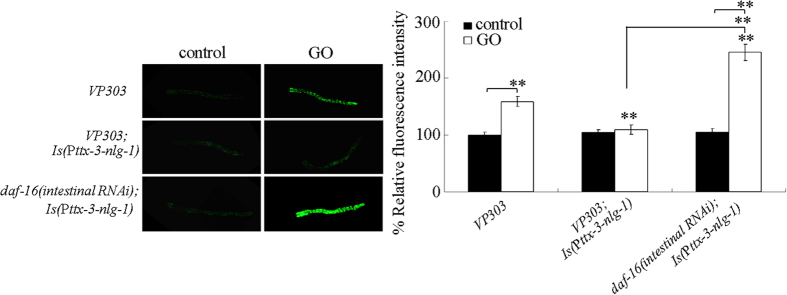
Genetic interaction between NLG-1 and DAF-16 in regulating response to GO exposure. Prolonged exposure was performed from L1-larvae to young adults. GO exposure concentration was 1 mg/L. Bars represent means ± SD. **P < 0.01 *vs* VP303 (if not specially indicated).

Previous study has demonstrated that the insulin signaling could function in the intestinal cells to regulate the GO toxicity^25^. In the insulin signaling pathway, *daf-16* gene encodes a FOXO transcriptional factor, and intestinal RNA interference (RNAi) knockdown of *daf-16* gene induced a susceptible property to GO toxicity^25^. Transgenic strain of VP303 is a powerful tool for the intestine-specific RNAi in nematodes^46^. Under the normal condition, we observed that overexpression of *nlg-1* gene in the AIY interneurons in VP303 did not induce the obvious induction of intestinal ROS production (Fig. 11). After GO (1 mg/L) exposure, VP303 with the overexpression of *nlg-1* gene in the AIY interneurons also did not show the noticeable induction of intestinal ROS production (Fig. 11), suggesting the resistant property of the transgenic strain of *Is*(P*ttx-3-nlg-1*) to GO toxicity. Moreover, we found that intestinal RNAi knockdown of *daf-16* gene caused the significant induction of intestinal ROS production in GO (1 mg/L) exposed transgenic strain of *Is*(P*ttx-3-nlg-1*) (Fig. 11), implying that the neuronal NLG-1 may act upstream of DAF-16 to regulate response to GO exposure.

## Discussion

In *C. elegans*, previous study has suggested that mutation of *nlg-1* gene could cause the deficits in a subset of sensory behaviors, such as thermosensation, sensory processing, and longevity^35^,^47^, implying that NLG-1 is required for the control of functions of neurons and longevity. In this study, we observed that GO exposure could potentially decrease the expression of NLG-1 (Fig. 1). Meanwhile, our previous studies have demonstrated that GO exposure could decrease the locomotion behavior and reduce the lifespan^17^,^23^. Therefore, the reduction of NLG-1 expression may serve as an important contributor to the observed GO toxicity.

Previous studies have suggested that mutation of *nlg-1* gene was sensitive to paraquat, a commonly used ROS generator^35^,^36^,^37^. In this study, using the intestinal ROS production and locomotion behavior as the endpoints, we found that mutation of *nlg-1* gene induced a susceptible property to GO toxicity (Fig. 2). It was reported that GO exposure could induce the activation of oxidative stress^17^,^23^. Compared with the intestinal ROS production in GO exposed wild-type N2 nematodes, the more severe induction of intestinal ROS production in GO exposed *nlg-1* mutant nematodes further supports the important role of NLG-1 in inducing the oxidative stress in GO exposed nematodes. Together, these results suggest that NLG-1 is required for the induction of both oxidative stress and stress response.

In *C. elegans*, NLG-1 is expressed in both the neurons and the muscle^35^. Because muscle-specific RNAi of *nlg-1* did not obviously affect the GO toxicity (data not shown), in this study, we focused on the examination of neuron-specific activity of NLG-1 in the regulation of response to GO exposure. For the neuron-specific activities of NLG-1 in the regulation of response to GO exposure, we found that the activity of NLG-1 in the AIY interneurons was required for the induction of GO toxicity in inducing intestinal ROS production and in decreasing locomotion behavior (Fig. 3). Nevertheless, not the activity of NLG-1 in all the interneurons was required for the induction of GO toxicity, since the expression of NLG-1 in the URB interneurons did not obviously affect the susceptible property of *nlg-1(ok259*) mutant to GO toxicity (Fig. 3). In this study, with the aid of *mec-7* promoter, we observed that the expression of NLG-1 in the PVD sensory neurons did not noticeably influence the susceptible property of *nlg-1(ok259*) mutant to GO toxicity (Fig. 3). In *C. elegans, mec-7* promoter can also drive the genes to be expressed in touch receptor neurons (ALML/R, AVM, PVM, and PLML/R)^48^, which implies that the activity of NLG-1 in touch sensory neurons may be not required for the induction of GO toxicity. In this study, we also observed that the expression of NLG-1 in the URA motor neurons did not significantly affect the susceptible property of *nlg-1(ok259*) mutant to GO toxicity (Fig. 3). Considering the fact that both VA and DA are only required for the control of locomotion behavior in nematodes^49^, we did not examine the activities of NLG-1 in these motor neurons.

In this study, we identified the PKC-1 as a downstream molecular target for NLG-1 in the AIY interneurons to regulate the response to GO exposure. At least three lines of evidence were raised about this. Firstly, after GO exposure, mutation of *nlg-1* gene could alter the expression pattern of *pkc-1* gene (Fig. 4). Secondly, mutation of *pkc-1* gene could induce a susceptible property to GO toxicity (Fig. 5), which was similar to those observed in *nlg-1* mutant nematodes. Thirdly, expression of *pkc-1* in the AIY interneurons could reverse the susceptible property of *pkc-1(ok563*) mutant to GO toxicity (Fig. 9). More importantly, mutation of *pkc-1* gene could suppress the resistant property to GO toxicity in transgenic strain overexpressing *nlg-1* gene in AIY interneurons (Fig. 6). Therefore, NLG-1 may potentially act upstream of PKC-1 in the AIY interneurons to regulate response to GO exposure.

In *C. elegans*, TTX-3 and CEH-23 control the cell fate specification of AIY interneurons^38^,^50^. PTP-3 contains extracellular immunoglobulin-like and fibronectin type III domains and functions in the cell adhesion and the maintenance of presynaptic differentiation^39^. The observed decrease in the expression of *ttx-3, ceh-23*, and *ptp-3* genes in GO exposed *nlg-1(ok259*) mutant also implies the possible formation of deficits in the differentiation and development of the AIY interneurons.

In this study, we further identified the potential targeted genes for *pkc-1* in the regulation of response to GO exposure. Previous studies have suggested that PKC-1 is required for the secretion of neuropeptides^41^. However, after GO exposure, mutation of *pkc-1* did not alter the expression patterns of *egl-3* and *egl-21* genes encoding proneuropeptide processing enzymes (Fig. 7a), which implies that the observed susceptible property to GO toxicity in *pkc-1* mutant was not due to the altered neuropeptide release. After GO exposure, mutation of *pkc-1* gene altered the expression of *lin-45* encoding a Raf protein (Fig. 7a), implying that the observed susceptible property to GO toxicity in *pkc-1* mutant was due to the altered function of ERK signaling. However, mutation of *pkc-1* gene did not affect the expression of *let-60* encoding a Ras protein (Fig. 7a), suggesting that not the function of entire ERK signaling pathway was altered in GO exposed *pkc-1* mutant. The GO toxicity in *lin-45* mutant was similar to that in *pkc-1* mutant (Fig. 7c,d). Moreover, *lin-45* gene mutation could suppress the resistant property of transgenic nematodes overexpressing PKC-1 in the AIY interneurons to GO toxicity (Fig. 8). Therefore, PKC-1 may function upstream of LIN-15 in the AIY interneurons in the regulation of response to GO exposure. Moreover, we observed that *pkc-1* mutation could decrease the *lin-45*, while *lin-45* gene could be significantly induced by GO exposure in wild-type N2 nematodes (Fig. 7a and b). These results imply that mutation of *pkc-1* may suppress the protection function of LIN-45-mediated ERK signaling against the GO toxicity in nematodes.

Our results further demonstrated that mutation of genes encoding the NLG-1-PKC-1-LIN-45 signaling pathway may affect the GO translocation. Compared with GO/Rho B exposed wild-type nematodes, we observed the increased accumulation of GO/Rho B in the pharynx, the intestine, the spermatheca, and the tail in *nlg-1, pkc-1*, or *lin-45* mutant nematodes (Fig. 10a). Meanwhile, a relatively equable distribution of Rho B fluorescence was formed in tissues of wild-type N2, *nlg-1(ok259*), *pkc-1(ok5630*), or *lin-45(sy96*) mutant nematodes (Fig. 10b), implying that no noticeable alteration in intestinal permeability was formed in the *nlg-1(ok259*), *pkc-1(ok5630*), or *lin-45(sy96*) mutant compared with wild-type N2 nematodes under the normal condition. It was reported that an elevated levels of oxidized proteins were formed in *nlg-1* mutant nematodes^35^. Therefore, the formed oxidative damage may induce a susceptible property of *nlg-1* mutant to environmental toxicants; however, the formed oxidative damage may be not enough to alter the intestinal permeability in *nlg-1* mutant nematodes under the normal condition. In contrast to this, after GO exposure, mutation of *nlg-1, pkc-1*, or *lin-45* gene may induce a susceptibility to GO toxicity, which in turn cause the enhanced distribution and translocation of GO in the tissues of nematodes.

Our previous study has identified the insulin signaling as an intestinal signaling to regulate the GO toxicity in nematodes^25^. In this study, the NLG-1 has been shown to mediate a neuronal signaling in the regulation of response to GO exposure. Moreover, we found that intestinal RNAi knockdown of *daf-16* gene encoding the FOXO transcriptional factor in the insulin signaling pathway suppressed the resistant property of transgenic strain overexpressing NLG-1 in the AIY interneurons to GO toxicity (Fig. 11), which suggests that DAF-16 may act downstream of NLG-1 to regulate the response to GO exposure. More importantly, this observation implies the possible existence of an important link between the neuronal NLG-1 signaling and the intestinal insulin signaling. That is, during the response to GO exposure, the neuronal NLG-1 signaling may regulate the functions of certain intestinal signaling pathways such as the intestinal insulin signaling pathway.

**Figure 12:**
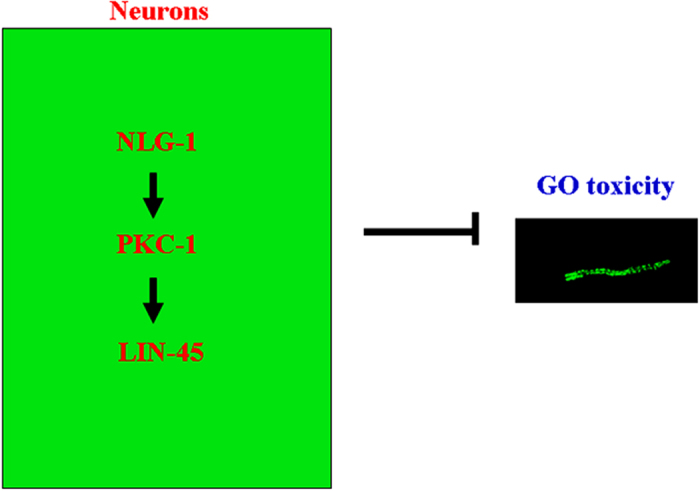
A diagram showing the role of neuronal NLG-1-PKC-1-LIN-45 signaling cascade in the induction of GO toxicity in nematodes.

In conclusion, using the *in vivo* assay system of *C. elegans*, we here investigated the effect of GO exposure on NLG-1-mediated signaling in neurons. In nematodes, GO exposure significantly decreased the NLG-1 expression, and mutation of *nlg-1* gene induced a susceptible property to GO toxicity, suggesting the crucial role of NLG-1 in the induction of GO toxicity. NLG-1 could act in certain interneurons such as the AIY interneurons to regulate the response to GO exposure. In the AIY interneurons, NLG-1 could function upstream of PKC-1, and PKC-1 further acted upstream of LIN-45 to regulate the response to GO exposure. Thus, the NLG-1-PKC-1-LIN-45 signaling cascade was raised in the AIY interneurons to be required for induction of GO toxicity (Fig. 12). Therefore, our results provide the important neuronal basis for the induction of GO toxicity in nematodes. Moreover, our data implies the important link between the neuronal NLG-1 signaling and the intestinal insulin signaling during the control of response to GO exposure.

In the clinical, mutations of human neuroligin 3 or neuroligin 4 gene are associated with autism spectrum disorders (ASDs)^51^,^52^. Moreover, it has been shown that certain mutations in human neuroligin 4 are also associated with other developmental disorders of the nervous system, such as X-linked mental retardation, Tourette syndrome, and schizophrenia^34^,^53^,^54^. The findings in this study implies that the related patients with the mutations of neuroligin 3 or neuroligin 4 gene may need to pay attention to the influences of environmental toxicants, such as the ENMs.

## Methods

### GO preparation and characterization

According to a modified Hummer’s method, the GO was prepared from a natural graphite powder^55^. In a 250-mL flask, graphite (2 g) and sodium nitrate (1 g) were first added, and the concentrated H_2_SO_4_ (50 mL) was then added on ice, followed by the addition of KMnO_4_ (7 g). After temperature of the mixture reached to 35 °C, H_2_O (90 mL) was slowly dripped and stirred at 70 °C for 15 min to dilute the suspension. After treatment with a mixture of 7 mL of 30% H_2_O_2_ and 55 mL of H_2_O, the suspension was then filtered in order to obtain a yellow-brown filter cake. After washing with a solution of 3% HCl, the filter cake was further dried at 40 °C for 24 h. Finally, the GO would be obtained after the ultrasonication of as-made graphite oxide for 1 h.

To determine the physicochemical properties, GO was characterized by Raman spectroscopy, atomic force microscopy (AFM), and zeta potential. To perform the AFM assay, GO suspension was pipetted onto the Si substrates, air-dried, and then examined under the AFM tip (SPM-9600, Shimadzu, Japan). The GO thickness was approximately 1.0 nm based on the AFM assay, which implied the one layer property for the prepared GO (Fig. S1a). Sizes of most of the examined GO after sonication (40 kHz, 100 W, 30 min) were in the range of 40–50 nm (Fig. S1b). Raman spectroscopy was examined using a 632 nm wavelength excitation (Renishaw Invia Plus laser Raman spectrometer, Renishaw, UK). Raman spectroscopy assay indicated that GO had a G band at 1594 cm^−1^ and a D band at 1335 cm^−1^ (Fig. S1c). Zeta potential was examined by a Nano Zetasizer (Malvern Instrument Ltd. UK) using a dynamic light scattering (DLS) technique. Zeta potential of GO (100 mg/L) in K-medium was −21.3 ± 2.1 mV.

### C. elegans strains and GO exposure

Nematodes used were wild-type N2, mutants of *nlg-1(tm1961), pkc-1(ok563), abi-1(tm494)*, and *lin-45(sy96)*, and transgenic strains of *Ex(Pnlg-1-nlg-1::GFP), nlg-1(tm1961)Ex(Pttx-3-nlg-1), nlg-1(tm1961)Ex(Pcfi-1-nlg-1), nlg-1(tm1961)Ex(Pflp-3-nlg-1), nlg-1(tm1961)Ex(Pmec-7-nlg-1), Ex(Pttx-3-nlg-1), pkc-1(ok563);Ex(Pttx-3-nlg-1), Ex(Pttx-3-pkc-1), lin-45(sy96);Ex(Pttx-3-pkc-1), pkc-1(ok563)Ex(Pttx-3-pkc-1), lin-45(sy96)Ex(Pttx-3-lin-45)*, VP303/*kbIs7[nhx-2p::rde-1], Is(Pttx-3-nlg-1)*, and *daf-16(intestinal RNAi);Is(Pttx-3-nlg-1)*. The wild-type and mutant strains were obtained from the *Caenorhabditis* Genetics Center (funded by NIH Office of Research Infrastructure Programs (P40 OD010440)). Nematodes were normally maintained at 20 °C as described^13^. After washing off from the plates into centrifuge tubes, the gravid nematodes were lysed with a bleaching mixture (0.45 M NaOH, 2% HOCl) to obtain the age synchronous L1-larvae populations as described^56^.

GO at the working concentrations (1, 10, and 100 mg/L) was prepared by diluting the stock solution (1 mg/mL) with K medium after sonication (40 kHz, 100 W, 30 min). Prolonged exposure was performed from L1-larvae to young adults, and GO exposure was performed in the wells of 12-well sterile tissue culture plates at 20 °C with the addition of food (OP50).

### Toxicity assessment of GO

The intestinal ROS production was used to reflect the functional state of intestinal cells^57^. The intestinal ROS production was analyzed as described previously^58^,^59^. The nematodes were transferred into 1 μM 5′,6′-chloromethyl-2′,7′-dichlorodihydro-fluorescein diacetate (CM-H2DCFDA; Molecular Probes) to incubate for 3 h at 20 °C in the dark. After that, the nematodes were mounted onto a 2% agar pad for the examination at 510 nm of emission filter and 488 nm of excitation wavelength under a laser scanning confocal microscope (Leica, TCS SP2, Bensheim, Germany). Relative intestinal fluorescence intensity was semi-quantified. After normalization to the intestinal autofluorescence, the semiquantified ROS signals were expressed as relative fluorescence units (RFU). Three replicates were performed, and twenty nematodes were examined per treatment.

The locomotion behaviors of head thrash and body bend were used to reflect the functional state of motor neurons in nematodes^60^. These locomotion behaviors were analyzed under the dissecting microscope by eyes as described^61^,^62^. The head thrash has been defined as a change for the bending direction at the mid body. The body bend has been defined as a change for the direction of the part corresponding to posterior bulb of pharynx along the *y* axis, assuming that nematodes were traveling along the *x* axis. Three replicates were performed, and twenty nematodes were examined per treatment.

### GO distribution and translocation assay

To examine the GO translocation and distribution, Rho B was loaded on the GO by mixing a Rho B solution (1 mg/mL, 0.3 mL) with an aqueous GO suspension (0.1 mg/mL, 5 mL) as described^45^. The unbound Rho B would be removed using dialysis against the distilled water over 72 h. The prepared GO/Rho B was stored at 4 °C. Nematodes were incubated with GO/Rho B for 3 h. After washing with three times of M9 buffer, the nematodes were analyzed under a laser scanning confocal microscope (Leica, TCS SP2, Bensheim, Germany). In this study, the Rho B staining alone was used as a control.

### Reverse-transcription and quantitative real-time polymerase chain reaction (qRT-PCR) assay

Total nematode RNAs were extracted using an RNeasy Mini kit (Qiagen). After the RNA extraction, the RNAs were reverse transcribed using a PrimeScript^TM^ RT reagent kit (Takara, Otsu, Shiga, Japan) to obtain the cDNA. The real-time qRT-PCR was performed using a SYBR Premix Ex Taq™ (Takara) for the aim of amplifying PCR products. Real-time qRT-PCR was run at an optimized annealing temperature of 58 °C. The relative quantification of certain targeted gene in comparison to the reference *tba-1* gene encoding a tubulin protein was determined. The final results would be expressed as the relative expression ratio between the certain targeted gene and the reference *tba-1* gene. Three replicates were performed. The related primer information for qRT-PCR is shown in Table S1.

### DNA constructs and germline transformation

To generate entry vector carrying promoter sequence, promoter region for *ttx-3* gene specially expressed in the AIY interneurons, *cfi-1* gene expressed in the URA neurons, *flp-3* gene expressed in the URB neurons, or *mec-7* gene expressed in the PVD neurons was amplified by PCR from wild-type *C. elegans* genomic DNA. These amplified promoter fragments were inserted into the pPD95_77 vector in the sense orientation. *nlg-1*/*C40C9.5e, pkc-1*/*F57F5.5a*, or *lin-45*/*Y73B6A.5b* cDNA was amplified by PCR, and further inserted into the corresponding entry vector carrying *ttx-3, cfi-1, flp-3*, or *mec-7* promoter. Germline transformation was performed by coinjecting a testing DNA (10–40 μg/mL) and a marker DNA of P*dop-1::rfp* (60 μg/mL) into the gonad of nematodes as described^63^. The related primer information for DNA constructs is shown in Table S2.

### RNAi assay

RNAi was performed by feeding nematodes with certain *E. coli* strain HT115 (DE3) expressing a double-stranded RNA that is homologous to the targeted gene of *daf-16* as described^64^. *E. coli* HT115 (DE3) was first grown in the LB broth containing ampicillin (100 μg/mL) at 37 °C overnight, and then plated onto the NGM plates containing ampicillin (100 μg/mL) and isopropyl 1-thio-β-D-galactopyranoside (IPTG, 5 mM). The L2 larvae were transferred onto the RNAi plates, and let to develop into the gravid. The gravid adults were further transferred onto a fresh RNAi plate to lay eggs for 2 h so as to obtain the second generation of RNAi population. The laid eggs were allowed to develop into the young adults for the subsequent assays.

### Statistical analysis

Data were expressed as means ± standard deviation (SD). We performed the statistical analysis using a SPSS 12.0 software (SPSS Inc., Chicago, USA), and the differences between the groups were further determined using an analysis of variance (ANOVA). The probability level of 0.05 or 0.01 was considered to be statistically significant.

## Additional Information

**How to cite this article**: Chen, H. *et al*. Graphene Oxide Dysregulates Neuroligin/NLG-1-Mediated Molecular Signaling in Interneurons in *Caenorhabditis elegans. Sci. Rep.* **7**, 41655; doi: 10.1038/srep41655 (2017).

**Publisher's note:** Springer Nature remains neutral with regard to jurisdictional claims in published maps and institutional affiliations.

## Supplementary Material

Supporting Information
