# Supplementary material for: Graphene Oxide Dysregulates Neuroligin/NLG-1-Mediated Molecular Signaling in Interneurons in Caenorhabditis elegans
Source: Sci Rep. 2017 Jan 27;7:41655. doi: 10.1038/srep41655 (PMC5269675; doi:10.1038/srep41655)
Supplement: Supporting Information [file srep41655-s1.doc]

**Graphene Oxide Dysregulates Neuroligin/NLG-1-Mediated** **Molecular** **Signaling in Interneurons in *Caenorhabditis elegans***

He Chen, Huirong Li & Dayong Wang^*^

Key Laboratory of Environmental Medicine Engineering in Ministry of Education, Medical School, Southeast University, Nanjing 210009, China

^*^Correspondence and requests for materials should be addressed to D.W. (e-mail: [dayongw@seu.edu.cn](mailto:dayongw@seu.edu.cn)).

**Supporting Information:**


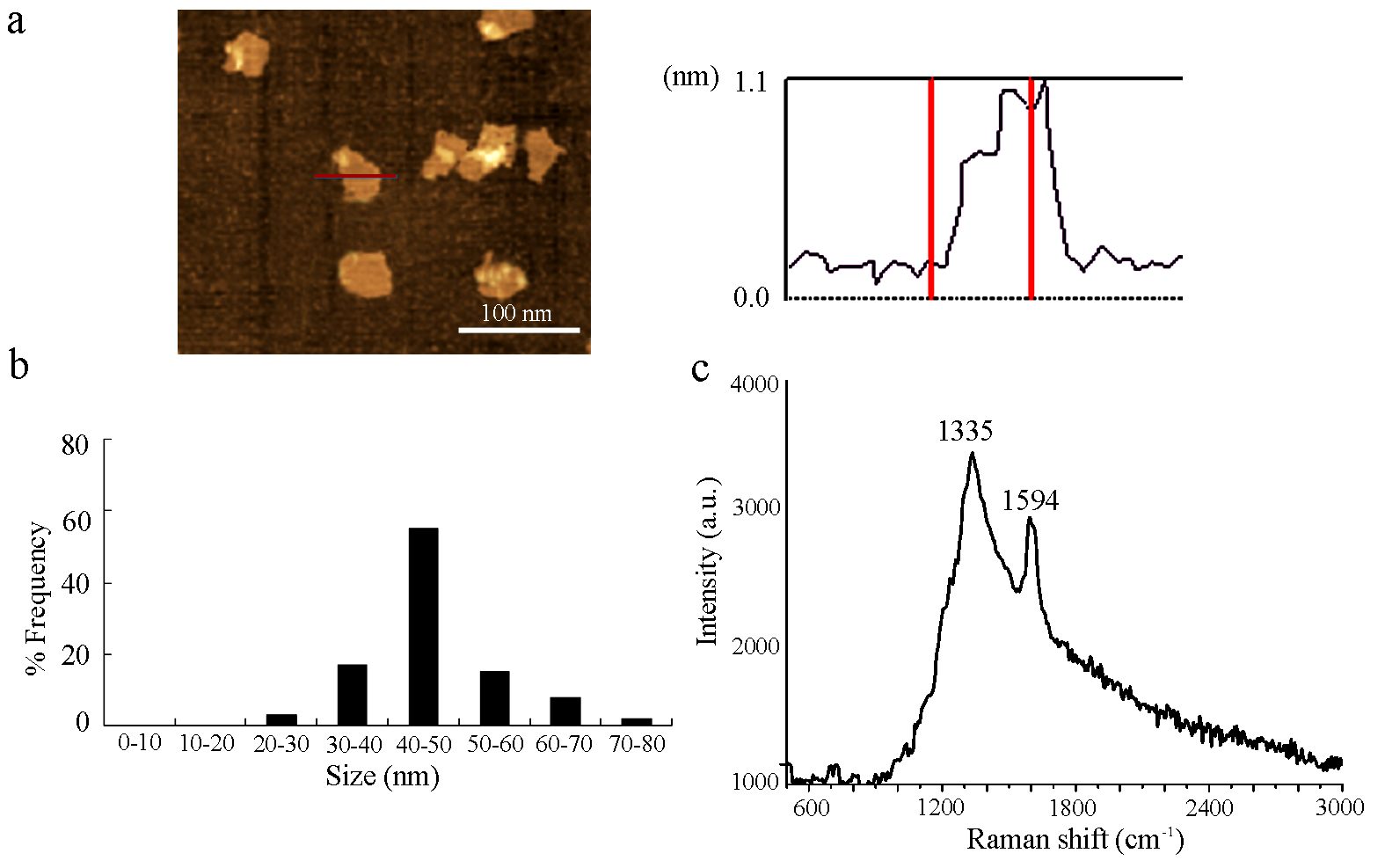


**Figure S1.** **Physiochemical properties of GO.** (**a**) AFM analysis of GO. (**b**) Size distribution of GO after sonication. (**c**) Raman spectrum of GO.

**Table S1.** **Primer information for qRT-PCR**

| Gene | Forward primer (5’-3’) | Reverse primer(5’-3’) |
| --- | --- | --- |
| *abi-1* | TCTGGCAGTTCTCCATCAC | GCATACGGTCTCATTGTTCTTC |
| *cam-1* | GTTTGGAGTTGTGCATTCTG | GCTCAAGTTGTGCTCTTTC |
| *ceh-10* | GTACGGCATTCTCTTCCACTT | ACCACGGAGCAGCACTTT |
| *ceh-23* | CAACCGTCACGCTTCAAGTC | TTCATAGCCAAGGGAACAGC |
| *ttx-3* | AACGGTGTTATGCGAAGAGGC | ATGCGTTTCGATCGTTGGCTT |
| *cnb-1* | AAAGACTCTCAACTCCAACAAAT | GTGTGCTCAACGACATCA |
| *dkf-2* | ACCGACGAAATGGTCAACAC | ACTTCAGCCCGCAACAGAT |
| *fax-1* | CTGAGAAGAGACAAATAATCCTAAC | TGGTCGTCGAAAGAGACAT |
| *cav-1* | ACGCCAACTCGATGAACA | ACGCAATCCCATGAGTGCT |
| *ptp-3* | TACAAACTGAAGAGCAATACATC | GTTCACCGCATCAAGGAC |
| *gcy-1* | TGGAACCAACCGAGTTAAGAAG | GACGTGATCCATGAGAGC |
| *hen-1* | CTTTGCTCATCCGTTTTG | AGTAGTTTTCGTCGTCTC |
| *inx-7* | GCCTGGTACATCTTGCT | GAAATGCTCATTGTAAGTCTCA |
| *inx-19* | GAGTCCAGCGAGTTTAAGAAG | CGCAAACGCAATGACAGTAT |
| *flp-1* | GGACGATCTCAACCAAAC | GACCGAAACGAAGGAAGT |
| *flp-18* | GTGTTCCAGGCGTTCTTC | TGGGATCTCCCGTTTGTC |
| *kal-1* | TCGACACCGAACTTCAAAC | GGACCTTCACACTGAACTG |
| *lin-35* | GCCAATTCTTTGCTGGTCTA | TTTCGGGATTCAGTTGTTCT |
| *ced-10* | TGCAAGCGATCAAATGTGTC | TGCGTTTGTGGTGTAGGA |
| *kin-29* | TTGCTCCATCTGCATCTTC | CTGTTCAGGTGTTGACATTCC |
| *lin-11* | CGCCGTGACGATATTGTAGAT | AGGTGGTGGGTAGAATTGTTG |
| *ncr-1* | GCGTCTTCGGCATCATTT | AAGCCGCTCCTTTCACATC |
| *ncs-1* | GAGAATAAGGATGGTGCTATA | TACACTACTTCCCACCATTTT |
| *pkc-1* | ACGCGACGCGAACAACTTTG | CGAACGACTGCAGGATCACTT |
| *ser-2* | GGTGGCGATATTCGTCAT | GCATACGGTAACTCCAAGA |
| *lin-53* | AGTGTGGCAAATGGCTGATA | CGGCTGGAGTTTCTTCGT |
| *cdc-42* | CTGCTGGACAGGAAGATTAC | GTCGGTCTGTGGATACGAT |
| *unc-9* | CACGACTACATTCCACATAAC | GCTTCAAGAGCTAACACAAAC |
| *unc-17* | TCATTGCCGGATGGATTGT | ACGTTGGTGGCGAAGATTA |
| *sra-11* | CCAGCGATTGACTGCAAGTATG | CCCGGCCAGTACTTCAAC |
| *bus-18* | CCTGTTGAACGAGGAGATGGAG | TTTCGTGATTCCTGCCACTTGA |
| *tax-6* | TGCGATTCGGGACATCTTAT | TGTTGGTGGCATTCTTTCA |
| *chd-3* | CAAAGCTCCTCGTTTACCA | CGAGCCACCTTCTCTTCATT |
| *set-2* | GCATCCGACTGCAATCTTC | CGTTGGAGCAATCTCATATCTT |
| *wrk-1* | GCTTTGCCACCGATTAGA | GACACTGTTGCAGTCCTC |
| *nlg-1* | TCTACCGCCAGTAGCACAGT | TGATTCACATCGTCCCAAAA |
| *let-60* | TACGACCCGACCATAGAGGACA | GACGCTCACGATGCTTGCGAAT |
| *lin-45* | AGGAGCAGGAACAAGTGACAAG | TGCCGCCCGCCTTTGTTCTGAACGC |
| *egl-3* | ATCTCGATCAATTCAGCCATTA | CAACTCCATTCATTTGCCATTC |
| *egl-21* | CCTTCCATCCATGAACCCAGAT | GGCAACCGAGTACCACTTAGCT |
| *tba-1* | TCAACACTGCCATCGCCGCC | TCCAAGCGAGACCAGGCTTCAG |

**Table S2.** **Primer information for vector constructions**

| Gene | Forward primer (5’-3’) | Reverse primer(5’-3’) |
| --- | --- | --- |
| P*ttx-3* | ATCTCTAGAATATGCACCCCGCTGACA | TGCGGATCCTTGAAAAGTAGGAAGCAT |
| P*cfi-1* | ATACTGCAGTAAACTTGTCGATTATTA | ATAGGATCCCTGCAAGAAAATACAAAC |
| P*flp-3* | ATACTGCAGAACTCCATCCTATCAATG | ATATCTAGATGGTGGTTATGGTGGTGT |
| P*mec-7* | CGCCTGCAGAGTAATCTAGAAATGTAA | ATAGGATCCGTTGCTTGAAATTTGGAC |
| P*nlg-1* | ATACTGCAGTAAGCCCCCGTACGCTAA | ATAGGATCCGCCTGTTCACTTCCAAAT |
| *nlg-1/C40C9.5e* | ATACCCGGGATGGAACGCATTTATCTT | GTGGAATTCATGCGAAAATAGTATACT |
| *pkc-1/F57F5.5a* | ATACCCGGGATGCTGTTCACAGGCACC | CGCGGTACCTGAAAAACCACGAAACTC |
| *lin-45/Y73B6A.5b* | ATACCCGGGATGTCTGCAATGGCTGTA | ATACCATGGCTAAATGAGACCATAGAC |
